# The novel TFEB agonist desloratadine ameliorates hepatic steatosis by activating the autophagy-lysosome pathway

**DOI:** 10.3389/fphar.2024.1449178

**Published:** 2024-09-18

**Authors:** Jieru Lin, Chunhuan Huang, Jingye Zhao, Lu Li, Zhenwei Wu, Tingyu Zhang, Yuyin Li, Wei Li, Baoqiang Guo, Zhenxing Liu, Aipo Diao

**Affiliations:** ^1^ School of Biotechnology, Tianjin University of Science and Technology, Tianjin, China; ^2^ School of Basic Medical Science, Inner Mongolia Medical University, Hohhot, Inner Mongolia, China; ^3^ Department of Life Sciences, Faculty of Science and Engineering, Manchester Metropolitan University, Manchester, United Kingdom

**Keywords:** desloratadine, TFEB agonist, autophagy, lysosome, hepatic steatosis, obesity

## Abstract

The autophagy-lysosome pathway plays an essential role in promoting lipid catabolism and preventing hepatic steatosis in non-alcoholic fatty liver disease (NAFLD). Transcription factor EB (TFEB) enhances the autophagy-lysosome pathway by regulating the expression of genes related to autophagy and lysosome biogenesis. Therefore, targeting TFEB provides a novel strategy for the treatment of lipid metabolic diseases. In this study, the antiallergic drug desloratadine was screened and identified as a novel TFEB agonist. Desloratadine effectively induced translocation of TFEB to the nucleus and promoted autophagy and lysosome biogenesis. Desloratadine-induced TFEB activation was dependent on AMPK rather than mTORC1. Moreover, desloratadine treatment enhanced clearance of lipid droplets in cells induced by fatty acids oleate and palmitate. Furthermore, high-fat diet (HFD) induced obesity mouse model experiments indicated treatment with desloratadine markedly reduced the body weight of HFD-fed mice, as well as the levels of hepatic triglycerides and total cholesterol, serum glutamic pyruvic transaminase and glutamic-oxaloacetic transaminase. Oil red O staining showed the liver fat was significantly reduced after desloratadine treatment, and H&E staining analysis demonstrated hepatocellular ballooning was improved. In addition, autophagy and lysosomal biogenesis was stimulated in the liver of desloratadine treated mice. Altogether, these findings demonstrate desloratadine ameliorates hepatic steatosis through activating the TFEB-mediated autophagy-lysosome pathway, thus desloratadine has an exciting potential to be used to treat fatty liver disease.

## 1 Introduction

Non-alcoholic fatty liver disease (NAFLD) is a common chronic liver disease characterized by the accumulation of fat in liver cells, it is caused by an imbalance in the uptake and elimination of free fatty acids (FFAs) in the liver (Anstee et al., 2013). NAFLD may progress from simple steatosis (fatty liver) to non-alcoholic steatohepatitis (NASH), which is a more severe form of the disease (Loomba et al., 2021). The primary intervention for NAFLD includes controlling risk factors such as obesity, diabetes and hyperlipidemia, and modifying lifestyle such as balance diet and fitting exercise (Sodum et al., 2021). Currently, there are limited therapeutic drugs available for the treatment of NAFLD, thus it is imperative to identify more precise targets and effective compounds for prevention and treatment of this disease with lipid metabolism defect.

Autophagy is a highly conserved process in which damaged organelles or dysfunctional macromolecules are degraded in lysosomes to maintain cellular homeostasis (Li et al., 2021). The dysregulation of the autophagy-lysosome pathway leads to the aggregation of macromolecules such as lipids and glycogen, ultimately resulting in impaired metabolism and intracellular stress (Martinez-Lopez and Singh, 2015; Yasmina et al., 2021). Recent research has demonstrated that the suppression of autophagy-lysosome pathway exacerbates hepatic steatosis and non-alcoholic steatohepatitis (Ren et al., 2024; Tanaka et al., 2016). Transcription factor EB (TFEB), a basic helix-loop-helix leucine zipper (bHLH-LZ) transcription factor, belongs to the MITF-TFE family of transcription factors, which plays a significant role in the regulation of the autophagy-lysosome pathway (Napolitano and Ballabio, 2016; Settembre et al., 2011). TFEB binds to the promoter regions of numerous autophagy-related genes or lysosome-related genes (Roczniak-Ferguson et al., 2012; Yasmina et al., 2021), thereby stimulating autophagosome formation or lysosome biogenesis (Sardiello et al., 2009). Recently, a study has revealed that the inhibition of TFEB and impairment of autophagy in the liver of patients with NAFLD led to the continuous accumulation of lipids in the liver (Qian et al., 2021), ultimately resulting in the development of end-stage liver disease (Zhang et al., 2018). Alternatively, overexpression of TFEB has a significant improvement on the fatty liver of diet-induced or genetically induced obese mice (Singh et al., 2009). Therefore, targeting TFEB and activating the autophagy-lysosome pathway provide a novel strategy for the treatment of NAFLD.

In this study, we have screened and identified desloratadine as a novel TFEB agonist, which promoted nuclear translocation of TFEB and activation of the autophagy-lysosome pathway. Desloratadine, an 8-chloro-6,11-dihydro-11-(4-piperidenyl)-5H-benzo [5,6] cycloheptyl [1,2-B] pyridine compound, has been clinically used to treat chronic urticaria and allergic rhinitis (Murdoch et al., 2003). Using a fatty acid-induced lipid accumulation cell model and a high-fat diet-induced hepatic steatosis mouse model, we showed that desloratadine treatment promoted lipid clearance and ameliorated hepatic steatosis. These findings provide a molecular mechanism for the activation of the autophagy-lysosome pathway by desloratadine, which can be potentially applied in the treatment of metabolic syndrome with lipid overload.

## 2 Materials and methods

### 2.1 Chemical reagents and antibodies

All of the chemicals were sourced from Sigma Aldrich (St.Louis, MO, United States) unless otherwise specified. L1300 FDA-approved drug library was purchased from Selleck, United States. Desloratadine (purity 99.98%) and Torin1 (purity 98.77%) were procured from Selleckchem (Houston, Texas, United States) and dissolved in dimethyl sulfoxide (DMSO) for concentrated storage. Compound C (purity 99.91%) purchased from MCE (NJ, United States). The anti-LC3B antibody (1:2500, L7543) was obtained from Sigma Aldrich. Cathepsin B (CTSB) (1:2000, ab190077) and LAMP-2 (1:2000, ab25631) were purchased from Abcam (Cambridge, United Kingdom). Antibodies against p62 (1:1000, 5114), TFEB (1:1000, 4240), phosphorylated AMPK (p-AMPK) (1:1000, 2535) and phosphorylated p70S6K (p-p70S6K) (1:1000, 9204) were acquired from Cell Signaling Technology (Danvers, Massachusetts, United States). Antibodies against phosphorylated Akt (p-Akt) (1:500, sc-514032) and β-actin (1:500, sc-47778) were procured from Santa Cruz Biotechnology (Santa Cruz, California, United States). The anti-histone H3 (1:1000, KM9005), horseradish peroxidase (HRP) conjugated goat anti-rabbit (1:5000, LK2001) and anti-mouse secondary antibodies (1:5000, LK2003) were purchased from Sungene Biotech (Tianjin, China).

### 2.2 Cell lines and cell culture

HeLa, HepG2 and L02 cell lines were obtained from the American Type Culture Collection (ATCC) and cultured in Dulbecco’s modified Eagle medium (DMEM, Gibco, Eggenstein, Germany) supplemented with 10% fetal bovine serum and 1% antibiotics under a 5% CO_2_ atmosphere at 37°C. All cells used for experiments were less than 15 generations.

### 2.3 Plasmid construction and stable cell lines

The plasmid construction of pTFEB-GFP or pGFP-LC3 was performed as previously described to generate HeLa cells stably expressing TFEB-GFP or GFP-LC3 (Li et al., 2019). Briefly, full-length human TFEB and LC3 cDNAs were amplified using PCR and cloned into pLVX-AcGFP-N1 and pLVX-AcGFP-C1 plasmids (Clontech, Palo Alto, California, United States), respectively, to generate the pTFEB-GFP and pGFP-LC3 transfection vectors. Lentiviral transfer plasmid and packaging plasmids were co-transfected into HEK293T cells using Lipofectamine 2000 (Invitrogen, Carlsbad, California) according to the manufacturer’s instructions. Viruses were collected 48 h post-transfection and cryogenically preserved. Cells were infected with these lentiviruses overnight in the presence of 8 μg/mL polybrene and selected in 1 μg/mL puromycin for 2 weeks.

### 2.4 Cell viability assay

The cell viability was assessed using the 3-(4,5-dimethylthiazol-2-yl)-2,5-diphenyl tetrazolium bromide (MTT) dye absorbance and expressed as a percentage of untreated cells. Cells were seeded at a density of 7 × 10^3^ cells per well in 96-well plates. After treatment with desloratadine for 24 or 48 h, MTT solution (20 μL, 0.5 mg/mL in PBS) was added and incubated at 37°C for 4 h. DMSO (200 μL/well) was added to fully dissolve the crystals, and then the absorbance of each well was measured at a wavelength of 490 nm using a microplate reader.

### 2.5 Nuclear and cytoplasmic fractionation

HepG2 or L02 cells were treated with desloratadine, lysed with NP-40 lysis buffer (10 mM Tris-HCl, 150 mM NaCl, 0.05% NP-40) containing EDTA-free protease inhibitors, vigorously shaken for 10 s and left on ice for 10 min. The lysates were then centrifuged at 14,000 rpm for 10 min to separate into cytoplasmic protein and precipitated nuclear protein fractions, which were further analyzed using Western blot.

### 2.6 RNA interference

Knockdown of TFEB protein was performed in HepG2 cells by expressing short hairpin RNA (shRNA) targeting TFEB. The shRNA construct was generated using pLKO.1-puro lentiviral vector. Lentivirus packaging was carried out as described above. The levels of TFEB knockdown were detected by Western blot analysis. A non-target shRNA vector was used as a negative control.

### 2.7 Western blot

Total cell lysates were prepared using RIPA lysis buffer (20 mM Tris-HCl, 150 mM NaCl, 1% NP-40, 1% sodium deoxycholate) containing a protease inhibitor cocktail (MCE, New Jersey, United States). Equal amounts of protein samples underwent SDS-PAGE before being transferred onto a PVDF membrane (Roche, Basel, Switzerland). After blocking with TBST containing 5% non-fat milk, membranes were incubated overnight at 4°C in dilutions containing specific primary antibodies. Following washing with TBST buffer the bands were incubated with secondary antibodies appropriately coupled to HRP for 2 h at room temperature. Immunoblotting was performed using chemiluminescent reagents and visualized using a chemiluminescence detection system (GE Healthcare, Little Chalfont, Buckinghamshire, United Kingdom). Quantitative analysis was conducted using ImageJ software (Wayne Rasband NIH Bethesda MD United States), and β-actin protein levels served as loading controls.

### 2.8 Lipid droplets accumulation cell model

Dissolved sodium oleate (OA) and sodium palmitate (PA) powders in 0.1 M NaOH to make 100 mM stock solutions, heated for complete dissolution. 1% BSA-containing culture medium was added and incubated at 37°C for 20 min to form stable OA-BSA and PA-BSA complexes (10 mM), mimicking natural fatty acid transport in blood. OA-BSA and PA-BSA (2:1 OA:PA ratio) were combined to produce lipid droplets inducer (Hairong et al., 2024; Jin et al., 2021). Solution was filtered through a 0.45 μm sterile membrane and stored at −20°C. HepG2 or L02 cells were cultured in 12-well plates, and pre-treated with 0.6 mM OA and PA or OA for 12 h, followed by desloratadine or DMSO treatment for 24 h. Cells were fixed in 4% paraformaldehyde and stained with Oil Red O (ORO) for 1 h, after counterstained with hematoxylin and imaged under a microscope. Images from at least three different fields per sample were acquired, and calculated using ImageJ software.

### 2.9 High-fat diet mice model

Animal experiments were performed in accordance with the National and Institutional Guidelines for Animal Care and Use. The animal experiments in this study received approval from the Ethics Committee of the Tianjin University of Science and Technology, Tianjin, China (2023-Shengwu-003). Male C57BL/6 mice aged four to 5 weeks old were randomly divided into six experimental groups after 1 week of acclimation (n = 5/group). The mice were fed either a normal diet (ND) or high-fat diet (HFD, 60% kcal fat, 20% kcal protein and 20% kcal carbohydrate) for 10 weeks. After 10 weeks, the mice continuously received an HFD or ND with or without desloratadine administration (4 mg/kg or 8 mg/kg) for further 10 weeks. Body weight measurement was taken weekly.

### 2.10 Triglyceride, total cholesterol, glutamic-pyruvic transaminase and glutamic-oxaloacetic transaminase assay

Triglyceride and total cholesterol content in hepatocytes were quantified using a Triglyceride or Total Cholesterol Quantification kit (ZCIBIO Technology, Shanghai, China) according to the manufacturer’s manual. Glutamic-pyruvic transaminase (GPT/ALT) and glutamic-oxaloacetic transaminase (GOT/AST) content from mouse serum were measured using a ALT and AST activity assay kit (Solarbio Science and Technology, Beijing, China).

### 2.11 Hematoxylin-eosin (H&E) and Oil red O staining

Liver tissues were fixed with 4% paraformaldehyde, and paraffin sections were used for H&E staining, while frozen sections were used for Oil red-O staining. The H&E staining solution was purchased from Servicebio (Wuhan, China) and applied according to the manufacturer’s instructions. For *in vitro* lipid staining, the Oil red-O working solution was prepared by diluting the Oil red-O stock solution (5 mg Oil red-O in 100 mL of isopropanol) at a ratio of 3:2 with ddH_2_O. Cells were fixed in 4% paraformaldehyde for 30 min and then incubated for 5 min in 60% isopropanol. Subsequently, the cells were incubated for 30 min with the Oil red O working solution and washed for 30 s in 60% isopropanol. The cells underwent four washes with ddH_2_O and counterstained with hematoxylin before acquiring images using an Olympus microscope.

### 2.12 RNA extraction and quantitative real-time PCR

Total RNA was extracted from cells or mouse tissues using the TRIzol^®^ Reagent (Life Technologies, California, United States), and reverse transcription was performed using the All-in-One First-Strand cDNA Synthesis SuperMix kit for qRT-PCR (TransScript. Beijing, China). mRNA levels were detected using quantitative real-time PCR analysis with SybrGreen qPCR Mastermix (DBI Bioscience. Shanghai, China), following the protocol of the reagent. The sequences of primers used for qRT-PCR were shown in Table 1.

**TABLE 1:** List of primers for qRT-PCR.

List of primers for qRT-PCR			
	Genes	Forward primer (5′ to 3′)	Reverse primer (5′ to 3′)
Human	*β-actin*	GTG​GCC​GAG​GAC​TTT​GAT​TG	AGT​GGG​GTG​GCT​TTT​AGG​ATG
	*LC3B*	ACCATGCCGTCGGAGAAG	ATC​GTT​CTA​TTA​TCA​CCG​GGA​TT
	*p62*	ATCGGAGGATCCGAGTGT	TGGCTGTGAGCTGCTCTT
	*LAMP2*	TGG​CTC​CGT​TTT​CAG​CAT​TG	CGC​TAT​GGG​CAC​AAG​GAA​GT
	*CTSB*	AAA​AGC​AGA​AAA​CAG​CTC​CGC	ATC​TTG​CGC​AGA​AAG​TTG​GC
	*CTSD*	GCA​AAC​TGC​TGG​ACA​TCG​CTT​G	GCC​ATA​GTG​GAT​GTC​AAA​CGA​GG
	*LIPA*	GCGGCGCTGCCAGAATG	ATC​TGC​CAG​CAA​GCC​ATG​TT
Mouse	*GAPDH*	TGT​GTC​CGT​CGT​GGA​TCT​GA	CCT​GCT​TCA​CCA​CCT​TCT​TGA​T
	*LC3B*	GACCGGCCTTTCAAGCAG	TGG​GAC​CAG​AAA​CTT​GGT​CT
	*p62*	TGG​GCA​AGG​AGG​AGG​CGA​CC	CCT​CAT​CGC​GGT​AGT​GCG​CC
	*LAMP2*	GAG​CAG​GTG​CTT​TCT​GTG​TCT	ACA​CCC​ACT​GCA​ACA​GGA​ATA
	*CTSB*	AAA​AAG​GCC​TGG​TTT​CAG​GT	GGG​AGT​AGC​CAG​CTT​CAC​AG
	*CTSD*	TCA​GGA​AGC​CTC​TCT​GGG​TA	CCC​AAG​ATG​CCA​TCA​AAC​TT
	*LIPA*	AAG​CTC​GCC​TGC​TTG​TAG​TG	TGG​AGT​TGC​ATC​GGG​AGT​G

### 2.13 Statistical analysis

All experiments were repeated at least three times, and data are presented as mean ± SD from three independent experiments. Statistical significance was determined using unpaired Student t-test or one-way analysis of variance (ANOVA) performed on GraphPad Prism version 8.4 software (GraphPad Software). Differences were considered statistically significant when P < 0.05.

## 3 Results

### 3.1 Desloratadine induces TFEB nuclear translocation

**FIGURE 1:**
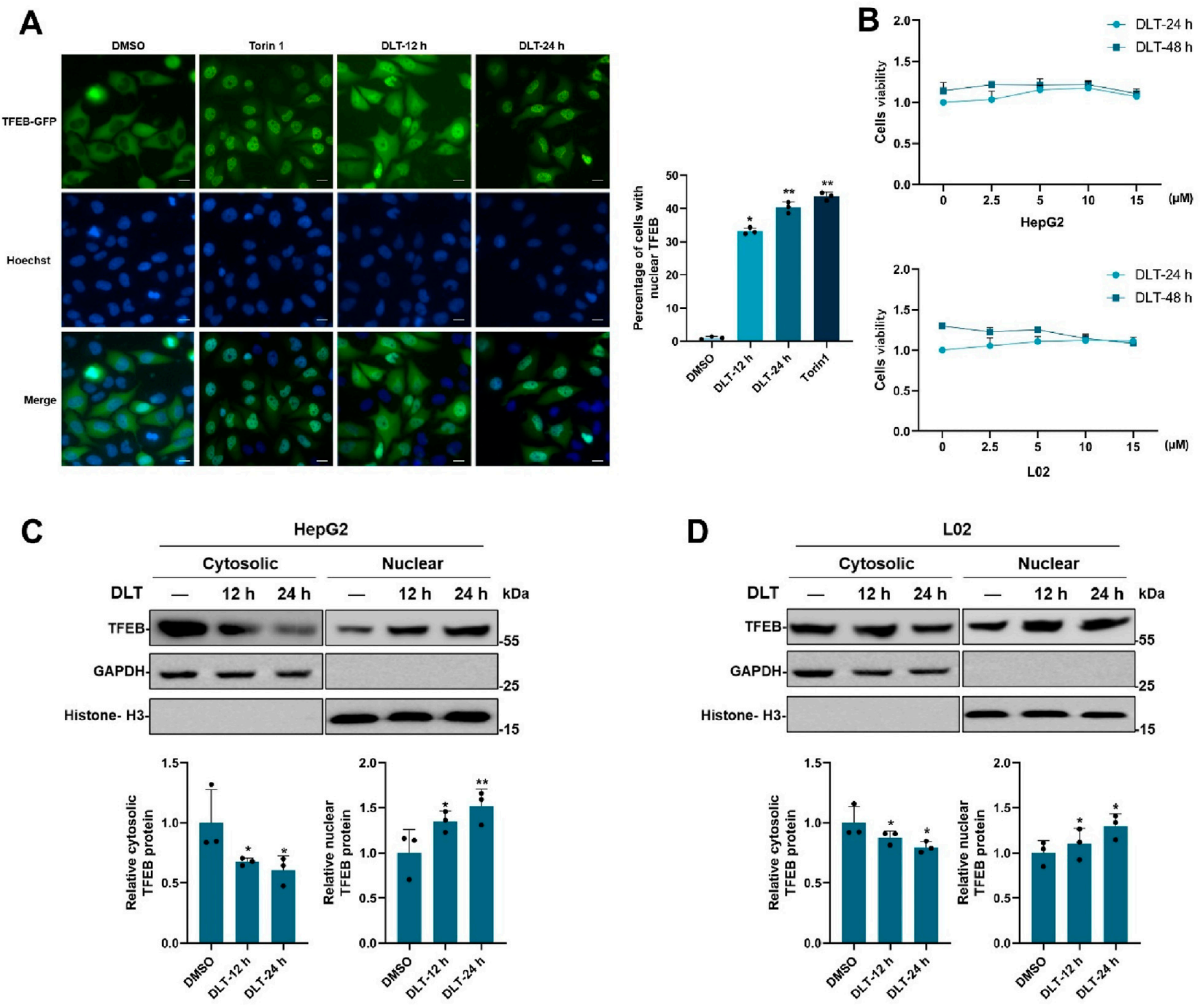
Desloratadine induces the nuclear translocation of TFEB. **(A)** Assessment of TFEB-GFP nuclear localization. HeLa cells expressing TFEB-GFP were treated with 10 μM desloratadine (DLT) for 12 h or 24 h, and observed using fluorescence microscopy. Nuclei were stained using Hoechst 33,342 (blue). Torin1 (200 nM, 1 h) was used as a positive control. Scale bar, 20 μm. The percent of cells with nuclear TFEB-GFP was quantified. **(B)** MTT assay for cells viability of HepG2 and L02 cells exposed to 0–15 μM DLT for 24 h or 48 h. **(C,D)** Effects of DLT on intracellular localization of endogenous TFEB. HepG2 and L02 cells were treated with or without 10 μM DLT. Western blot was used to detect endogenous TFEB protein levels in the nuclear and cytosolic fractions. GAPDH and Histone H3 were used as the loading controls. Lower panels indicate densitometric analysis of Western blot using ImageJ software. The protein levels of TFEB were normalized to levels of the cytosolic marker GAPDH or nuclear marker histone H3. Data are presented as mean ± SD of three independent experiments (*P < 0.05, **P < 0.01).

To screen for small molecule agonists of TFEB, HeLa cells stably expressing TFEB-GFP were produced and used for high-throughput screening. By screening a 3,067 compounds library set (FDA-approved drug library), desloratadine was found to potentially induced TFEB-GFP nuclear translocation. As showed in Figure 1A, 10 μM desloratadine treatment for 12 h or 24 h induced significant TFEB-GFP nuclear translocation under a fluorescence microscope, compared to the DMSO control. Similar results were observed in response to the known mTORC1 inhibitor Torin1 (200 nM, 2 h). The cytotoxicity of desloratadine against human hepatoma HepG2 cells and L02 cells was further assessed using an MTT assay and indicated that treatment with less than 15 μM desloratadine for 24 h did not show cytotoxicity (Figure 1B). Moreover, the nuclear translocation of endogenous TFEB in response to desloratadine treatment was evaluated by cytosolic and nuclear protein fractionation assay. The protein levels of nuclear TFEB in HepG2 and L02 cells were significantly increased after 10 μM desloratadine treatment (Figures 1C, D). These results suggest that desloratadine promotes TFEB nuclear translocation.

### 3.2 Desloratadine promotes autophagy

**FIGURE 2:**
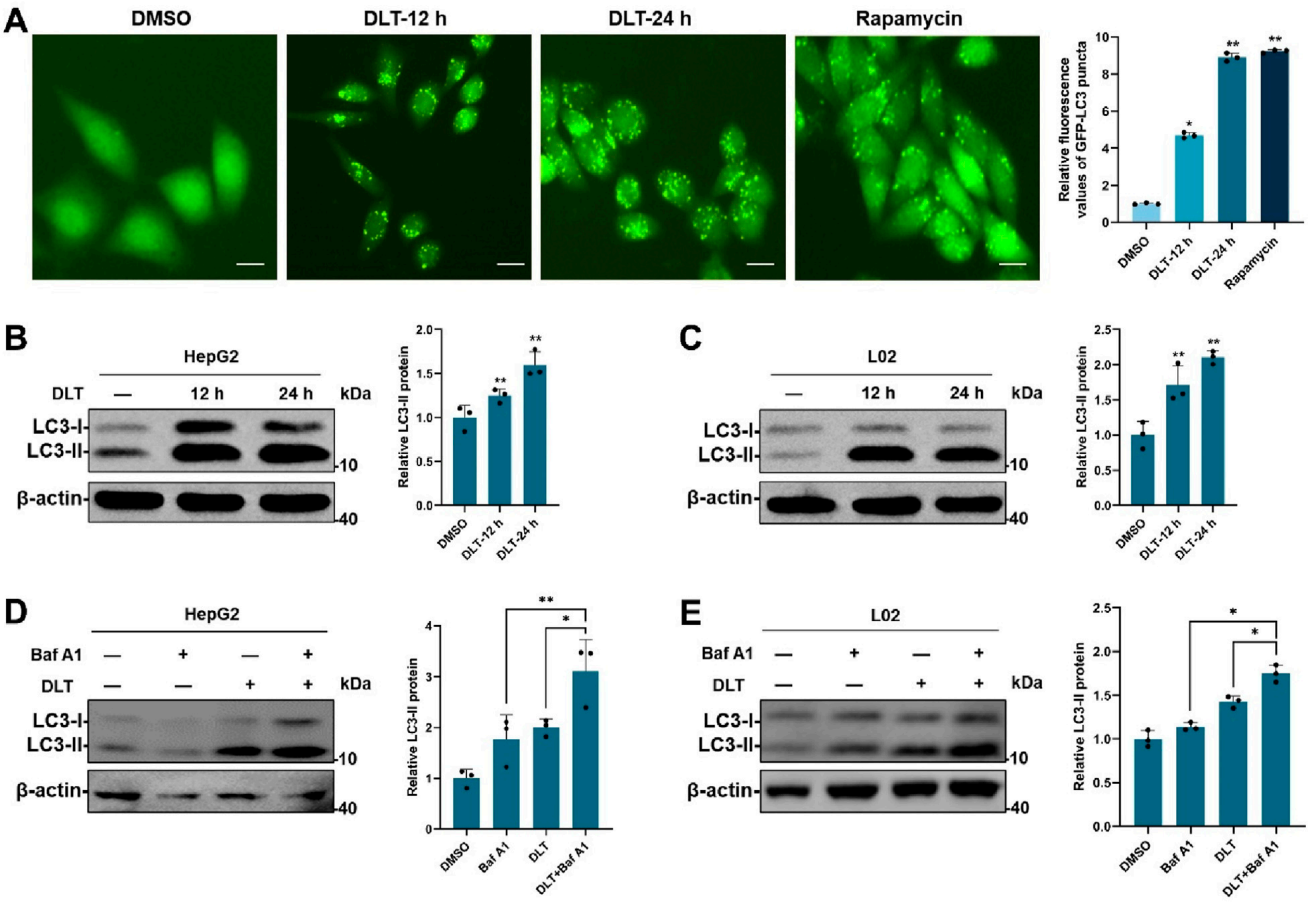
Desloratadine induces autophagy. **(A)** HeLa cells stably expressing GFP-LC3 were treated with 10 μM desloratadine (DLT) for 24 h or 48 h. The fluorescent signals of GFP-LC3 were sequentially acquired by fluorescence microscopy. Cells treated with rapamycin (Rapa, 200 nM, 12 h) were used as a positive control. Scale bar, 20 μm. The percentage of cells with GFP-LC3 puncta (≥5 dots) was quantified. **(B,C)** Effects of DLT on the protein levels of LC3-II. HepG2 and L02 cells were treated with 10 μM DLT for 12 h or 24 h respectively. The levels of LC3 were measured by Western blot using antibodies against LC3. β-actin was used as a loading control. **(D,E)** Following 10 μM DLT treatment for 12 h, the cells were treated with or without 200 nM Baf A1 for an additional 2 h. LC3 protein levels were detected by Western blot. LC3-II expression was quantified using ImageJ analysis and represented as the mean band intensity normalized to β-actin. Data are presented as mean ± SD of three independent experiments (*P < 0.05, **P < 0.01).

Given that TFEB is tightly linked to autophagy-lysosome activation, we then detected the roles of desloratadine in regulating autophagy. HeLa cells stably expressing GFP-LC3 were used to investigate the effect of desloratadine on autophagy induction. The fluorescence detection showed that desloratadine treatment (10 μM, 12 h or 24 h) markedly increased the number of GFP-LC3 puncta (Figure 2A), indicating the formation of autophagosomes in response to desloratadine treatment. Consistently, Western blot analysis demonstrated that the protein level of LC3-II was significantly increased in desloratadine-treated HepG2 and L02 cells, compared to those in DMSO-treated cells (Figures 2B, C). Autophagy flux involves autophagosome formation, cargo sequestration, and fusion with lysosomes for degradation. To gain a deeper understanding of the impact of desloratadine on autophagic flux, we employed a combined treatment strategy involving desloratadine and bafilomycin A1 (Baf A1). Baf A1 inhibits autophagic degradation by blocking lysosomal acidification, and is commonly used to assess autophagic flux (Daniel et al., 2021). Compared to treatment with Baf A1 or desloratadine alone, combined treatment with Baf A1 and desloratadine led to an accumulation of LC3-II, indicating that desloratadine enhances autophagic flux (Figures 2D, E). These results suggest that desloratadine promotes autophagy.

### 3.3 Desloratadine enhances lysosome function

**FIGURE 3:**
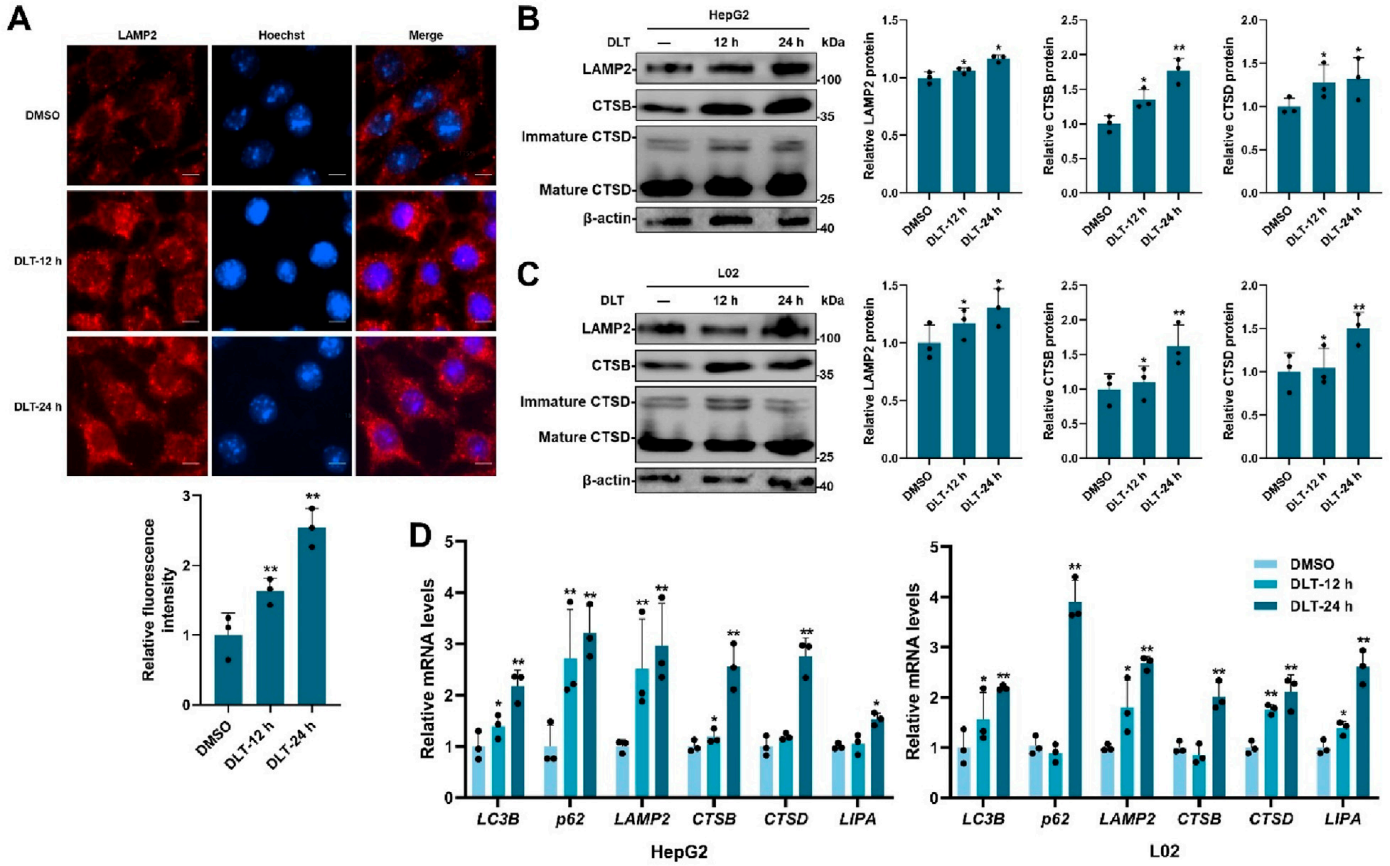
Desloratadine promotes lysosomal function. **(A)** HepG2 cells were treated with 10 μM desloratadine (DLT) for 12 h or 24 h, and then lysosomes were analyzed by immunofluorescence using antibody against LAMP2 (red fluorescence). The relative fluorescence intensity was analyzed using ImageJ software. Nuclear were stained with DAPI (blue). Scale bar, 20 μm. **(B,C)** Expression of LAMP2, CTSB and CTSD in 10 μM DLT treated HepG2, L02 cells were measured by Western blot. Quantitative analysis of the immunoblotted proteins was performed using ImageJ and represented as the mean band intensity normalized to β-actin. **(D)** The mRNA levels of *LC3B, p62, LAMP2, CTSB, CTSD* and *LIPA* in 10 μM DLT treated HepG2, L02 cells were detected by qRT-PCR analysis. Data are presented as mean ± SD of three independent experiments (*P < 0.05, **P < 0.01).

TFEB plays a key role in regulating lysosomal biogenesis [8–11]. We next measured the potential role of desloratadine in promoting lysosomal function. The fluorescence detection showed that desloratadine treatment (10 μM, 12 h or 24 h) significantly increased the number of lysosome associated membrane protein 2 (LAMP2) puncta, indicating a distinct increase in the number of lysosomes (Figure 3A). In addition, Western blot analysis showed increased protein levels of LAMP2, CTSB (cathepsin B) and CTSD (cathepsin D) in the desloratadine-treated HepG2 and L02 cells (Figures 3B, C). To further verify the effect of desloratadine on the transcriptional activity of TFEB, qRT-PCR was performed and demonstrated that the mRNA levels of *LC3, p62, LAMP2, CTSB, CTSD* and *LIPA* (lipase A) were significantly increased following desloratadine treatment (Figures 3D). These results suggest that desloratadine promotes lysosomal biogenesis.

### 3.4 Desloratadine upregulates AMPK signaling

**FIGURE 4:**
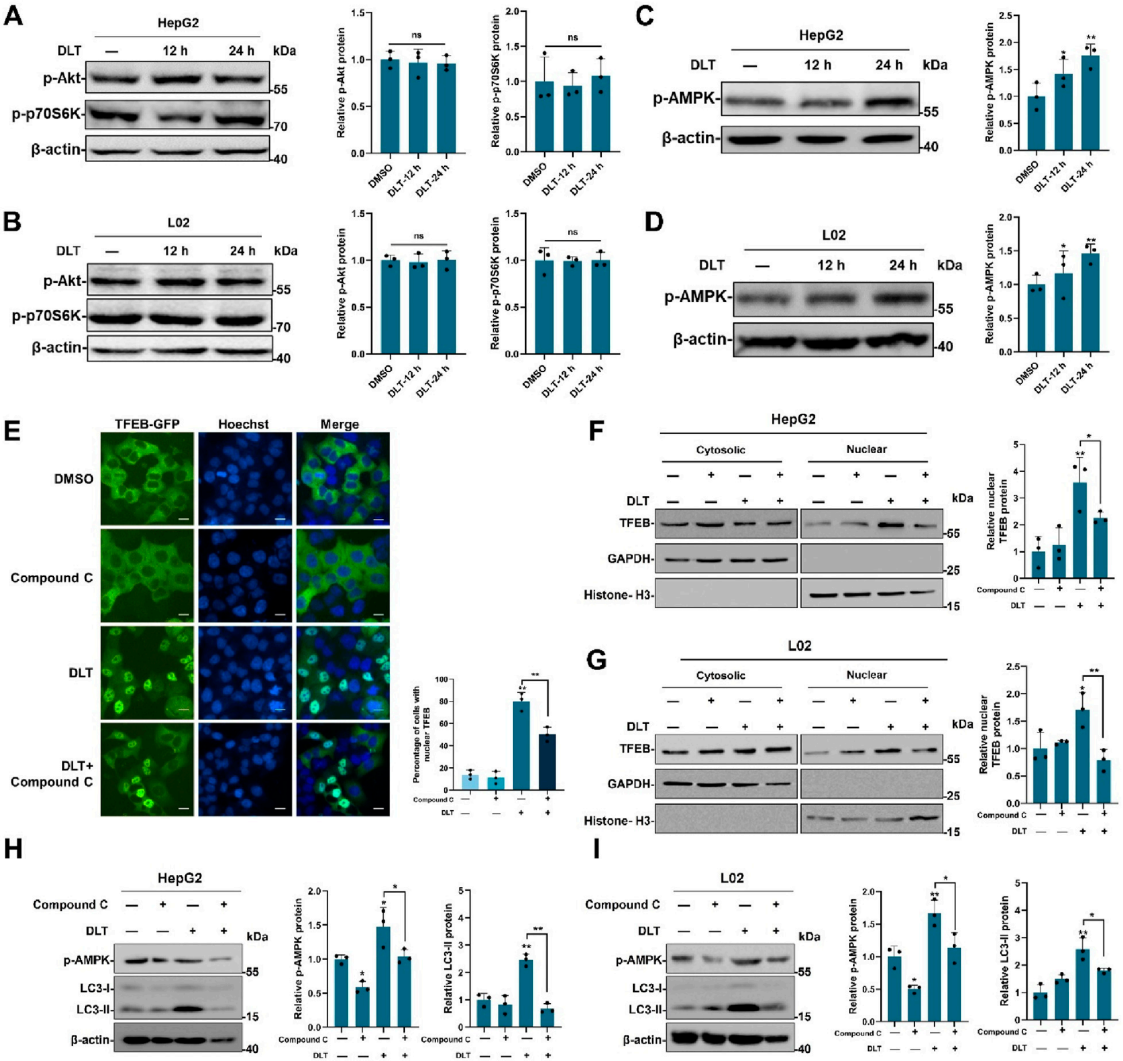
Desloratadine enhances the level of p-AMPK. **(A,B)** After treatment with 10 μM desloratadine (DLT) for 12 h or 24 h, HepG2 and L02 cells were subjected to Western blot analysis using the antibodies against p-Akt and p-p70S6K. β-actin was used as loading control. **(C,D)** The levels of p-AMPK were detected in the same desloratadine treated cells, and quantified by densitometric analysis and normalized to β-actin. **(E)** Assessment of TFEB-GFP nuclear localization. HeLa cells expressing TFEB-GFP were treated with Compound C (5 μM) and desloratadine (DLT) (10 μM) for 24 h and observed using a fluorescence microscope. Nuclei were stained with Hoechst 33,342 (blue). Scale bar, 20 μm. The percentage of cells with nuclear TFEB-GFP was quantified. **(F,G)** Effects of AMPK Inhibition on DLT-Induced nuclear translocation of TFEB. HepG2 and L02 cells were subjected to individual treatments with Compound C, Desloratadine (DLT), or a combination of both. Western blot was used to detect TFEB protein levels in nuclear and cytosolic fractions. GAPDH and histone H3 were used as loading controls. Densitometric analysis of Western blots was performed by ImageJ software. The protein level of TFEB was normalized to the level of cytoplasmic marker GAPDH or nuclear marker histone H3. **(H,I)** Western blot was used to measure the LC3-II levels after DLT treatment in presence of Compound C. β-actin was used as loading control. Data are presented as mean ± SD of three independent experiments (*P < 0.05, **P < 0.01, ns, not significant).

The serine/threonine kinases Akt-mTORC1 and AMPK signaling pathways are involved in nuclear translocation of TFEB, we then examined the effect of desloratadine on Akt-mTORC1 and AMPK activity. Firstly, the phosphorylation of Akt and p70S6K (a direct target of the mTORC1) were detected. As shown in Figures 4A, B, the levels of phosphorylated Akt (p-Akt) and p70S6K (p-p70S6K) were not affected in the desloratadine-treated HepG2 and L02 cells, indicating that mTORC1 activity was not repressed by desloratadine. Moreover, the level of phosphorylated AMPK (p-AMPK) was efficiently upregulated in response to desloratadine treatment (Figures 4C, D). In order to delve deeper into the potential association between DLT-mediated TFEB nuclear translocation and AMPK kinase activity, we employed the use of Compound C, an inhibitor of AMPK. The results revealed that treatment with Compound C significantly reversed the DLT-induced nuclear translocation of TFEB (Figures 4E–G). Additionally, the concurrent treatment of HepG2 and L02 cells with Compound C and DLT resulted in a substantial reduction in LC3-II levels (Figures 4H, I), which indicated desloratadine-induced TFEB translocation is dependent of AMPK signaling pathway.

### 3.5 Desloratadine promotes clearance of lipid droplets via TFEB activation

**FIGURE 5:**
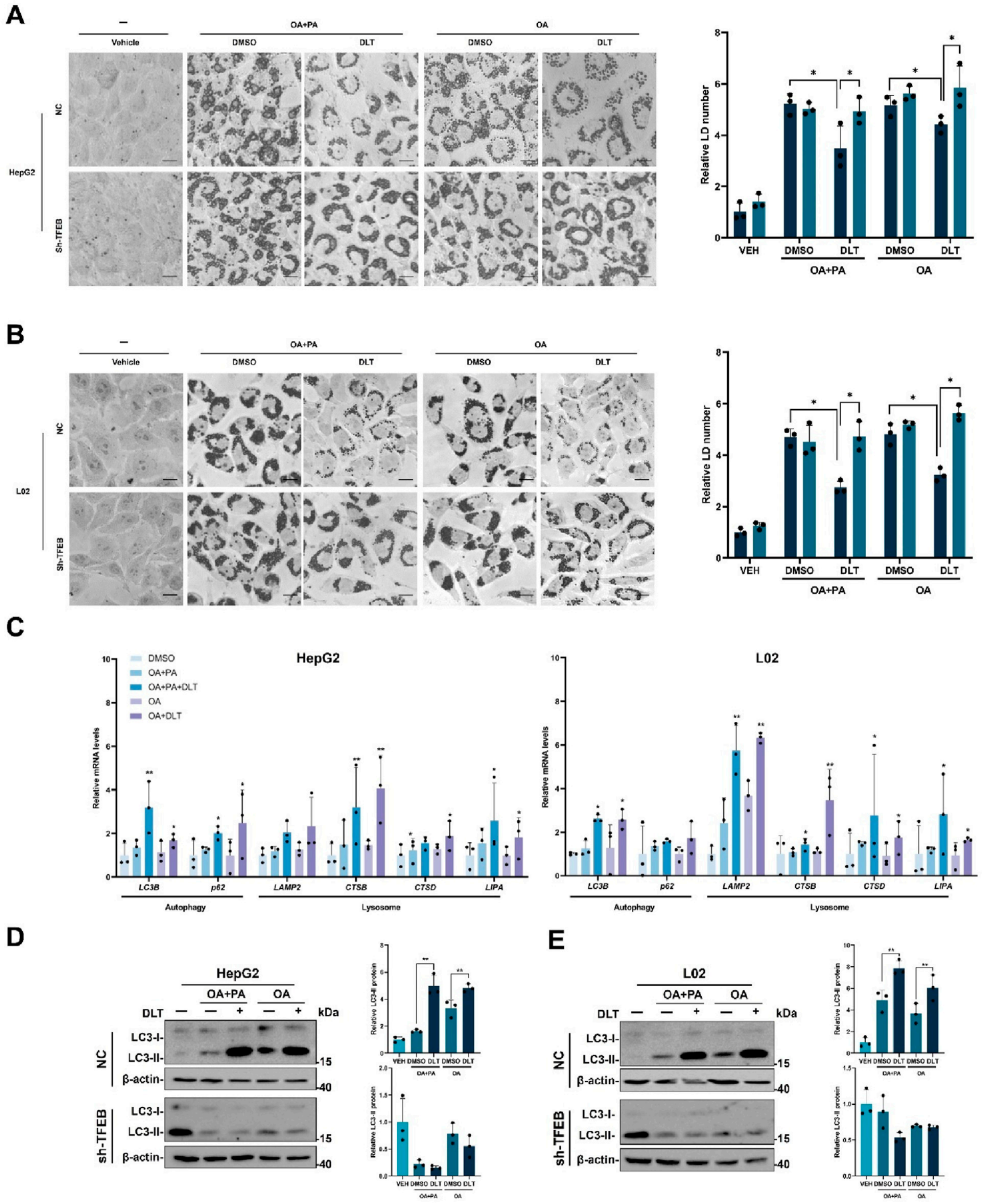
Desloratadine promotes clearance of lipid droplets. **(A,B)** HepG2 or L02 cells stably expressing negative control shRNA (NC) or TFEB shRNA (shTFEB) were pre-treated with 0.6 mM OA and PA or 0.6 mM OA alone respectively for 12 h, followed by stimulation with desloratadine (DLT) (10 μM) for 24 h. Lipid droplets were stained with oil red O (ORO). Scale bar, 20 μm. The accumulation of lipid droplets was quantified by densitometric analysis of staining intensity using ImageJ software. **(C)** The mRNA levels of *LC3B, p62, LAMP2, CTSB, CTSD* and *LIPA* in HepG2 and L02 cells co-treated with desloratadine (DLT) and OA plus PA or OA alone were detected by qRT-PCR analysis. **(D,E)** The LC3-II levels were detected by Western blot in HepG2 or L02 cells with co-treat desloratadine (DLT) and OA plus PA or OA alone. Quantified and normalized to β-actin. Data presented are the mean ± SD of three independent experiments (*P < 0.05, **P < 0.01).

It has been reported that lipid catabolism is regulated by the autophagy-lysosome pathway. We then investigated the potential of desloratadine in promoting lipid degradation using a fatty acid-induced lipid accumulation cell model. In HepG2 or L02 cells, exposure to either a combination of oleic acid (OA) and palmitic acid (PA) or OA alone triggered the formation of lipid droplets. Subsequent Oil Red O (ORO) staining revealed that treatment with desloratadine effectively mitigated both the quantity and size of the lipid droplets induced by either OA and PA or OA alone (Figures 5A, B). However, shRNA-mediated knockdown of TFEB attenuated the desloratadine-induced clearance of lipid droplets (Figures 5A, B). In addition, we examined the expression of autophagy-lysosome-related genes in the cells and found that DLT treatment significantly enhanced the expression of autophagy-lysosome genes in cells treated with OA and PA, or OA alone (Figure 5C). The level of LC3-II was also markedly elevated in DLT-treated cells. However, in the TFEB knockdown cells, DLT treatment failed to promote the expression of LC3-II (Figures 5D, E), indicating that desloratadine-induced activation of TFEB is involved in lipid clearance. These results suggest that desloratadine promotes clearance of lipid droplets by activation of TFEB as a master regulator of autophagy and lysosome biogenesis.

### 3.6 Desloratadine promotes liver fat catabolism and improves high-fat diet-induced hepatic steatosis by inducing autophagy-lysosome activation

**FIGURE 6:**
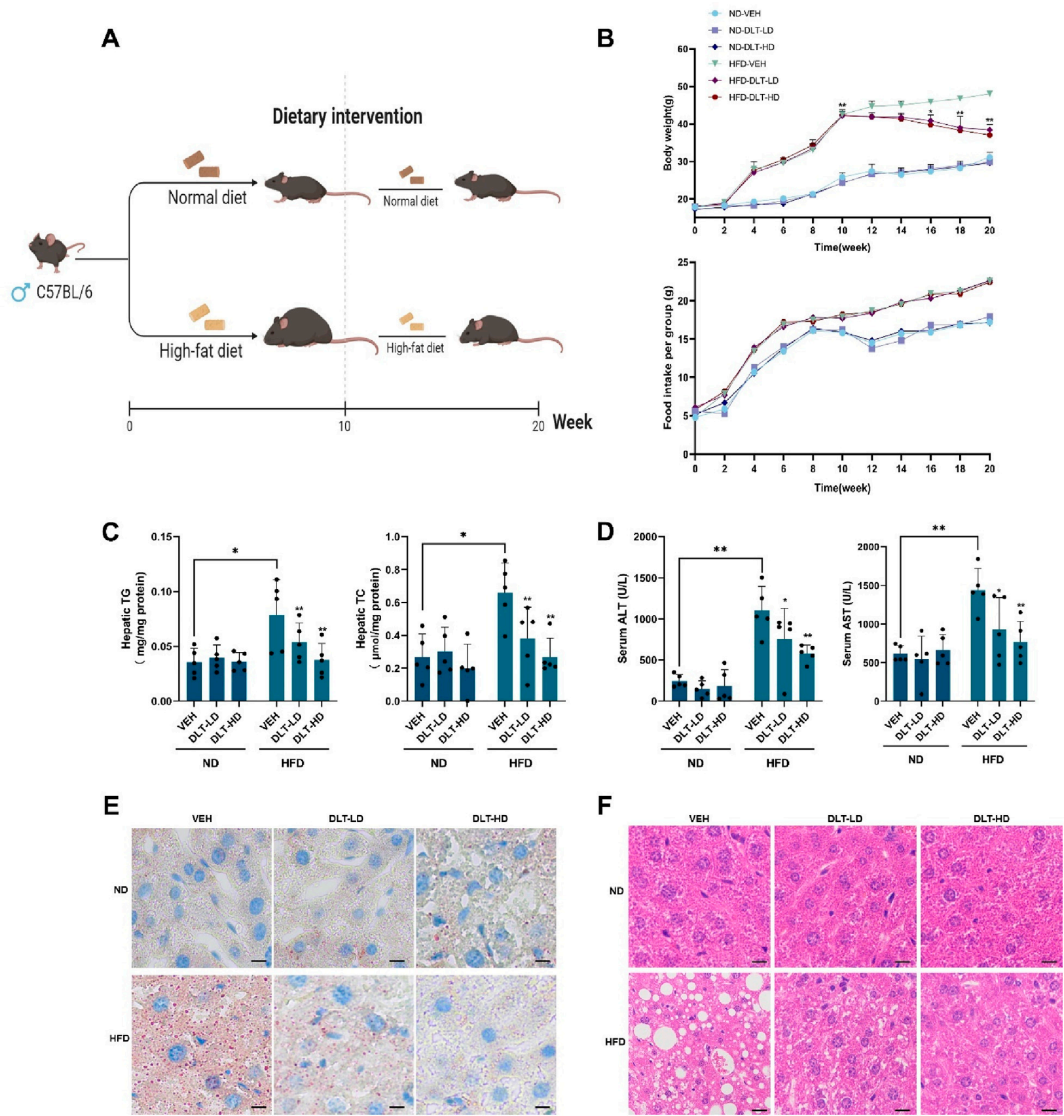
Desloratadine treatment ameliorates hepatic steatosis induced by a high-fat diet. **(A)** Schedule of animal treatments and experimental protocols. Male C57BL/6 mice were fed using a normal diet (ND) or high-fat diet (HFD) for 10 weeks (5 mice per group). After 10 weeks, mice were administered intraperitoneally (i.p.) with desloratadine (DLT) or vehicle (0.9% normal saline) once every other day for 10 weeks. Desloratadine (DLT) treatment groups were divided into low does (DLT-LD) of 4 mg/kg body weight, high does (DLT-HD) of 8 mg/kg body weight. **(B)** Mice body weight was measured biweekly. Food intake was checked once a week, the data was only presented every 2 weeks. **(C)** The levels of triglycerides (TG) and total cholesterol (TC) in liver tissue were detected by ELISA assay. **(D)** The levels of serum glutamic pyruvic transaminase (ALT) and glutamic-oxaloacetic transaminase (AST) were measured using activity assay kit. **(E)** Representative Oil red O stained images of liver morphology. Scale bar, 50 μm. **(F)** Representative hematoxylin and eosin (H&E) stained images of liver morphology. Scale bar, 50 μm. Data are presented as mean ± SD of three independent experiments (*P < 0.05, **P < 0.01).

A high-fat diet-induced hepatic steatosis mouse model was used to assess the effects of the identified TFEB agonist desloratadine on lipid clearance (Figure 6A). Firstly, C57BL/6 mice were fed a high-fat diet (HFD) for 10 weeks to develop an obese mouse model of NAFLD, and the normal diet (ND) fed mice were used as control. The daily dose of desloratadine in humans is up to 20 mg (Samir et al., 2002), equally about 4 mg/kg/day in mice. In this study, the mice were then administered with or without desloratadine (4 or 8 mg/kg) intraperitoneally every other day for further 10 weeks. Treatment with desloratadine markedly reduced the body weight of HFD-fed mice, compared to the untreated controls, and the weight loss achieved with desloratadine treatment reached ∼20% of the control (∼48 g) (Table 2; Figure 6B). We also monitored the food intake of mice and found that there was no obvious difference in food intake (Figure 6B). Importantly, desloratadine treatment had no effect on the ND-fed mice, indicating that desloratadine was not cytotoxic to the mice. Hepatic biochemical assays showed that desloratadine treatment significantly decreased triglycerides (TG) and total cholesterol (TC) levels in the liver of HFD-fed mice compared to the vehicle control (Figure 6C). Elevated levels of glutamic pyruvic transaminase (GPT/ALT) and glutamic-oxaloacetic transaminase (GOT/AST) indicate liver injury, HFD markedly increased AST and ALT levels. However, desloratadine treatment significantly attenuated the serum ALT and AST levels in the HFD-fed mice (Figure 6D). Furthermore, ORO staining analysis indicated that the amount of liver fat was significantly reduced after desloratadine treatment (Figure 6E), and H&E staining analysis demonstrated that hepatocellular ballooning was obviously improved in desloratadine treated HFD-fed mice (Figure 6F).

**TABLE 2:** Body weight of desloratadine-treated mice.

Body weight of desloratadine-treated mice			
Week	Group	Normal diet (g)	High-fat diet (g)
0	—	17.67 ± 1.13	17.94 ± 1.1
10	VEH	25.84 ± 1.54	42.28 ± 2.38
	DLT-LD	24.32 ± 1.48	42.15 ± 0.95
	DLT-HD	24.24 ± 0.56	42.37 ± 1.73
20	VEH	31.12 ± 2.22	48.05 ± 1.08
	DLT-LD	29.86 ± 1.84	38.42 ± 2.28
	DLT-HD	29.54 ± 0.76	37.03 ± 0.67

**FIGURE 7:**
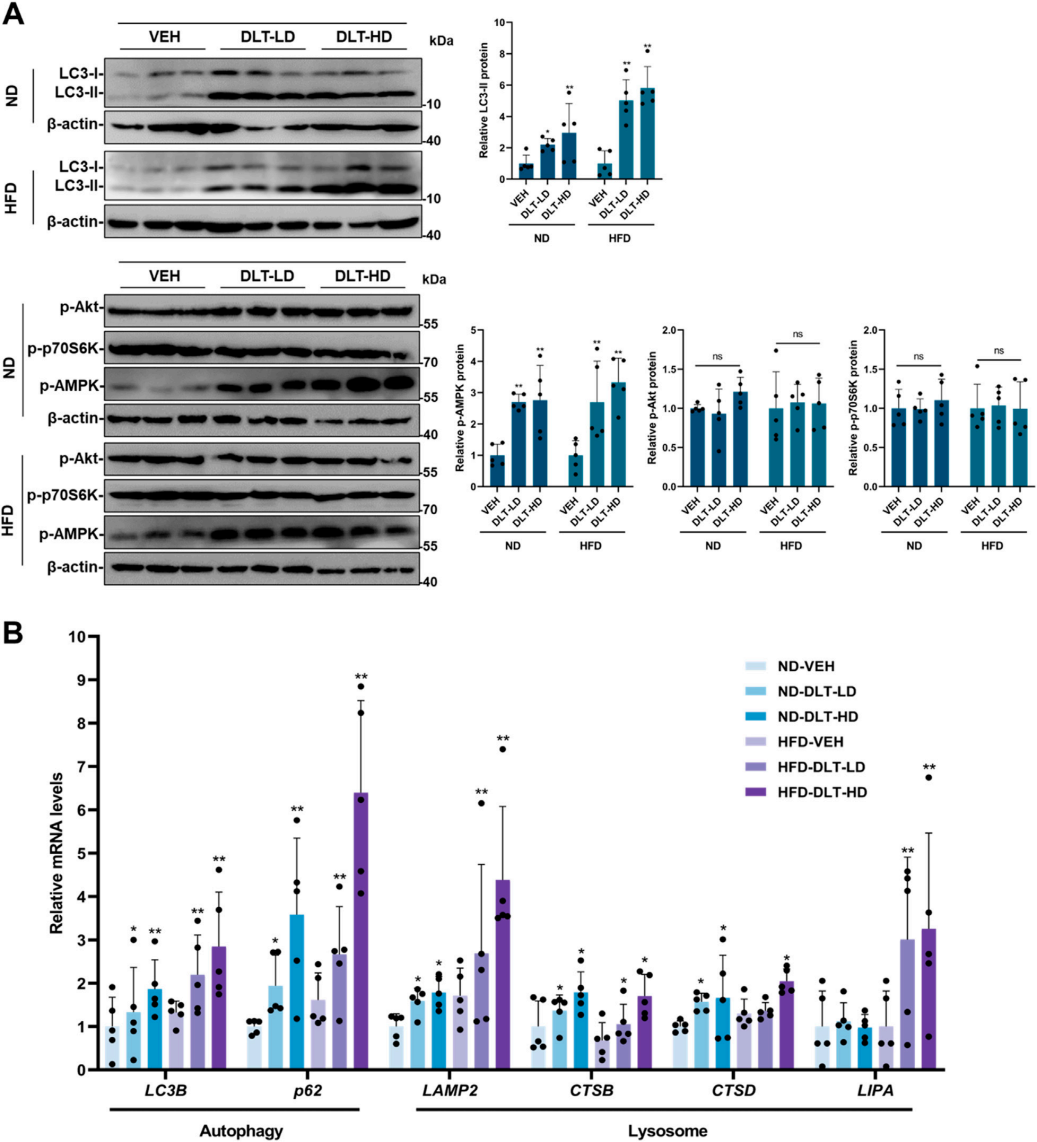
The autophagy-lysosome pathway is activated in the liver of mice treated with desloratadine. **(A)** The protein levels of LC3, p-AMPK, p-Akt and p-p70S6K in the liver tissue were assessed by Western blot. Blots were quantified and normalized to β-actin (n = 5). **(B)** The mRNA levels of *LC3B, p62, LAMP2, CTSB, CTSD* and *LIPA* in liver tissue were detected by qRT-PCR analysis (n = 5). Data are presented as mean ± SD of three independent experiments (*P < 0.05, **P < 0.01).

In addition, we investigated whether desloratadine could activate the autophagy-lysosome pathway *in vivo*. Western blot analysis showed that the protein levels of LC3-II and p-AMPK were significantly increased in liver tissues of both ND-fed and HFD-fed mice treated with desloratadine (Figure 7A). Moreover, qRT-PCR indicated that desloratadine treatment indeed upregulated the expression of genes associated with autophagy and lysosomal function, which included *LC3B, p62, LAMP2, CTSB, CTSD* and *LIPA* (Figure 7B). Collectively, these results suggest that desloratadine reduces hepatic steatosis through promoting autophagy-lysosome activation.

## 4 Discussion

Lysosomes play an essential role in macromolecule metabolism by degrading the contents delivered in the autophagosomes (Ueno and Komatsu, 2017). As the impairment of the autophagy-lysosome pathway has been mechanistically linked to metabolic disorders, such as NAFLD, boosting this innate intracellular clearance system provides a promising strategy for the treatment of these diseases (Du et al., 2022). Recent studies have demonstrated that the activation of TEFB by small-molecule agonists promotes the autophagy-lysosome pathway and ameliorates metabolic syndrome in the nematode *Caenorhabditis elegans* or mice (Lim et al., 2018; Wang et al., 2017). Our study demonstrates that desloratadine promotes lysosomal degradation machinery via activating TFEB and promotes lipid clearance in hepatic cells.

Lysosomal acid lipase (LAL), encoded by the gene *LIPA*, is the sole neutral lipid hydrolase in lysosomes and responsible for cleavage of triglycerides and cholesteryl esters. LAL is ubiquitously expressed with highest expression levels in hepatocytes and macrophages (Grabner et al., 2021). Lower LAL activity is associated with fatty liver disease (Besler et al., 2022). LAL defective mice have massive accumulation of triglycerides and cholesteryl esters in the liver, intestine, adrenal glands and macrophages (Du et al., 2001). Defect of LAL activity in humans also leads to severe hepatic steatosis and hepatosplenomegaly (Grabner et al., 2021). It has been reported that transcriptional regulation of LAL activity involves transcription factors TFEB, TFE3, FOXO1, PPARs (Zechner et al., 2017). Treatment of this newly identified TFEB agonist desloratadine leads to a decrease in accumulation of lipid droplets and liver fat, and the mRNA level of *lipase A (LIPA)* was increased in both desloratadine-treated cells and mice. These data suggest that desloratadine-induced clearance of lipids appears to occur through TFEB-mediated expression of LAL.

Desloratadine is an antihistamine and widely used to relieve allergy symptoms since being approved by FDA in 2001. As a second-generation antihistamine, desloratadine has been shown to be safe and well tolerated at nine times the recommended dose, 5 mg orally once a day (Bachert and Maurer, 2010). In this study, we elucidated the additional pharmacological properties of desloratadine, considering desloratadine boasts safety and a low incidence of side effects, which makes it suitable for long-term treatment, often necessary for chronic diseases such as hepatic steatosis. As a histamine H_1_ receptor antagonist, desloratadine binds with high affinity to the H_1_ receptor, and has been developed for treatment of allergic rhinitis (Philippe et al., 2008). Recently report indicated that desloratadine increased the expression of AMPK and showed proautophagy effect in glioblastoma cells (Sasenka et al., 2023). In this study, we showed that desloratadine treatment upregulates AMPK signaling in HepG2 cells and mouse liver.

It is known that mTORC1 directly phosphorylates TFEB at the serine 211 residue, and represses the activation of TFEB by maintaining its cytosolic sequestration through interaction with 14-3-3 protein (Martina et al., 2012; Roczniak-Ferguson et al., 2012). Although inhibiting mTORC1 signaling activates TFEB, mTORC1 is not an ideal drug target due to its other crucial roles in regulating cell growth and protein synthesis (Villa et al., 2021). In this study, Desloratadine did not inhibit the phosphorylation of p70S6K, an mTORC1 substrate, indicating that a mTORC1-independent process is responsible for the activation of TFEB induced by desloratadine. Interestingly, we found that desloratadine treatment induced the phosphorylation of AMP protein kinase (AMPK), which suggests that AMPK is involved in desloratadine induced TFEB activation. It has been reported that inhibition of mTORC1 by AMPK leads to the nuclear translocation of TFEB (Young et al., 2016). Another study showed that AMPK directly phosphorylates TFEB at S466, S467 and S469, which is essential for transcriptional activation of TFEB (Paquette et al., 2021). Recently a study showed that Mitochondrial damage-activated AMPK phosphorylates folliculin-interacting protein 1 (FNIP1), leading to dissociation of TFEB from RagC and mTORC1, therefore, TFEB is not phosphorylated and translocates to the nucleus (Malik et al., 2023). Our results indicate that desloratadine-mediated TFEB transcriptional activation is AMPK-dependent and mTORC1-independent. Therefore, further studies will elucidate the mechanism of desloratadine induced TFEB activation through regulating AMPK.

In summary, this study demonstrates that desloratadine promotes clearance of lipids and ameliorates hepatic steatosis through TFEB-mediated autophagy-lysosome pathway activation. These findings not only provide important mechanistic insights into the effect of desloratadine on activation of autophagy-lysosome pathway, but also suggest desloratadine as a novel TFEB agonist with potential for use in the treatment of lipid metabolic disorders, such as fatty liver disease, obesity.

## Data Availability

The original contributions presented in the study are included in the article/Supplementary Material, further inquiries can be directed to the corresponding authors.
