# Supplementary figures and images for: The novel TFEB agonist desloratadine ameliorates hepatic steatosis by activating the autophagy-lysosome pathway
Source: Front Pharmacol. 2024 Sep 18;15:1449178. doi: 10.3389/fphar.2024.1449178 (PMC11445182; doi:10.3389/fphar.2024.1449178)

The raw data of the Western Blots Images

Figure 1.

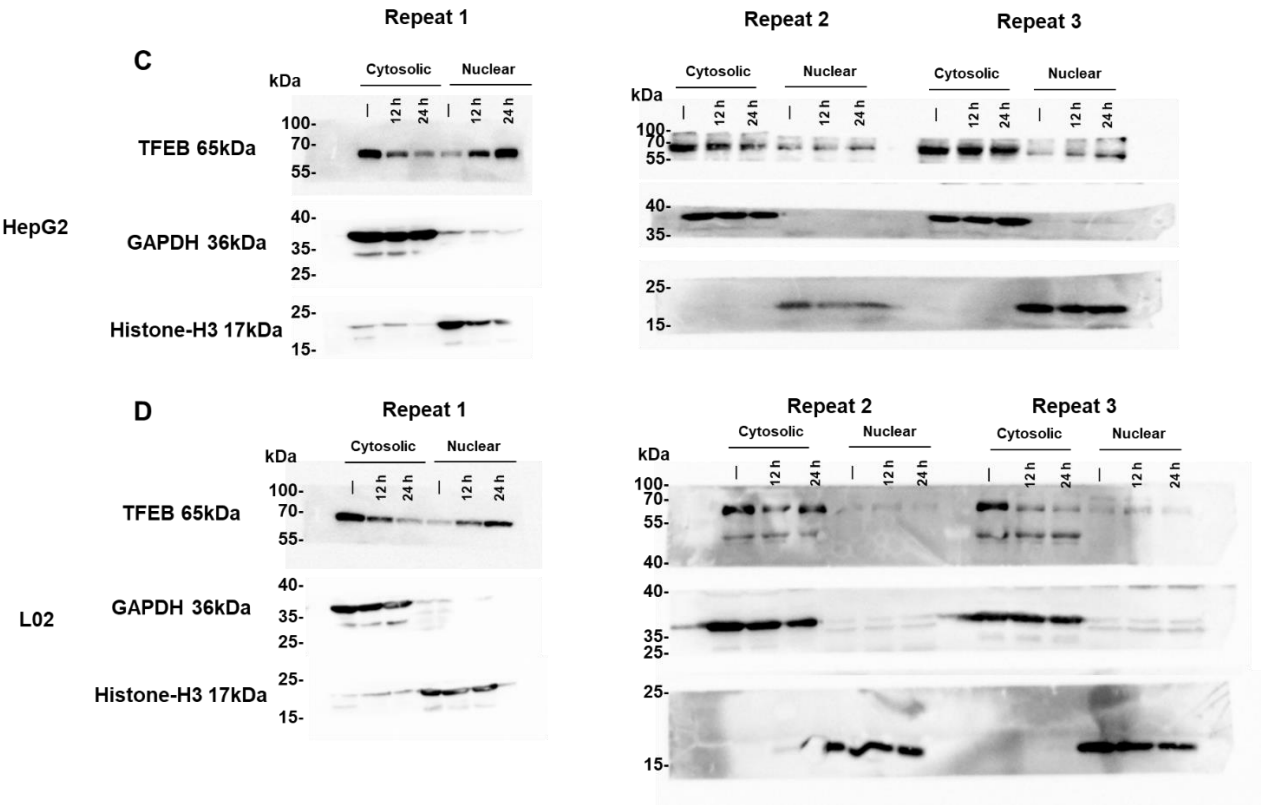

**Figure 2.**

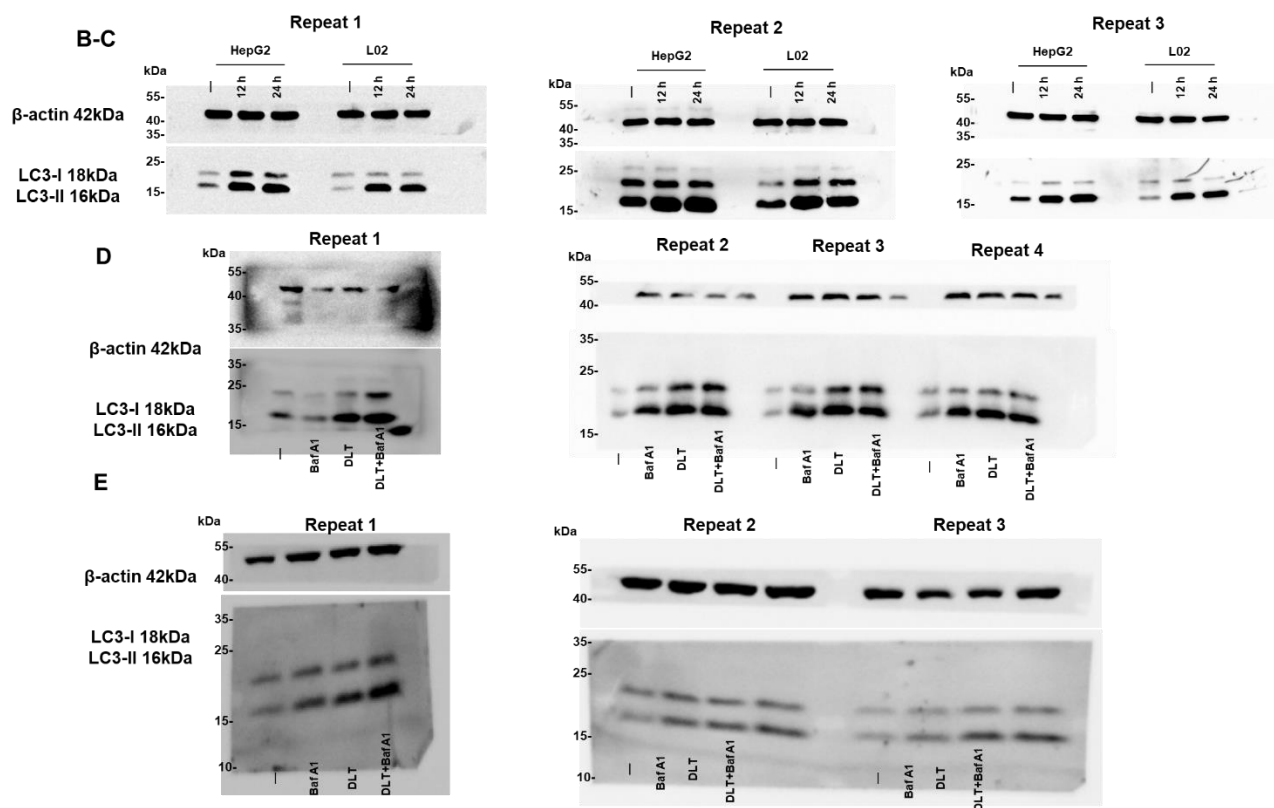

Figure 3.

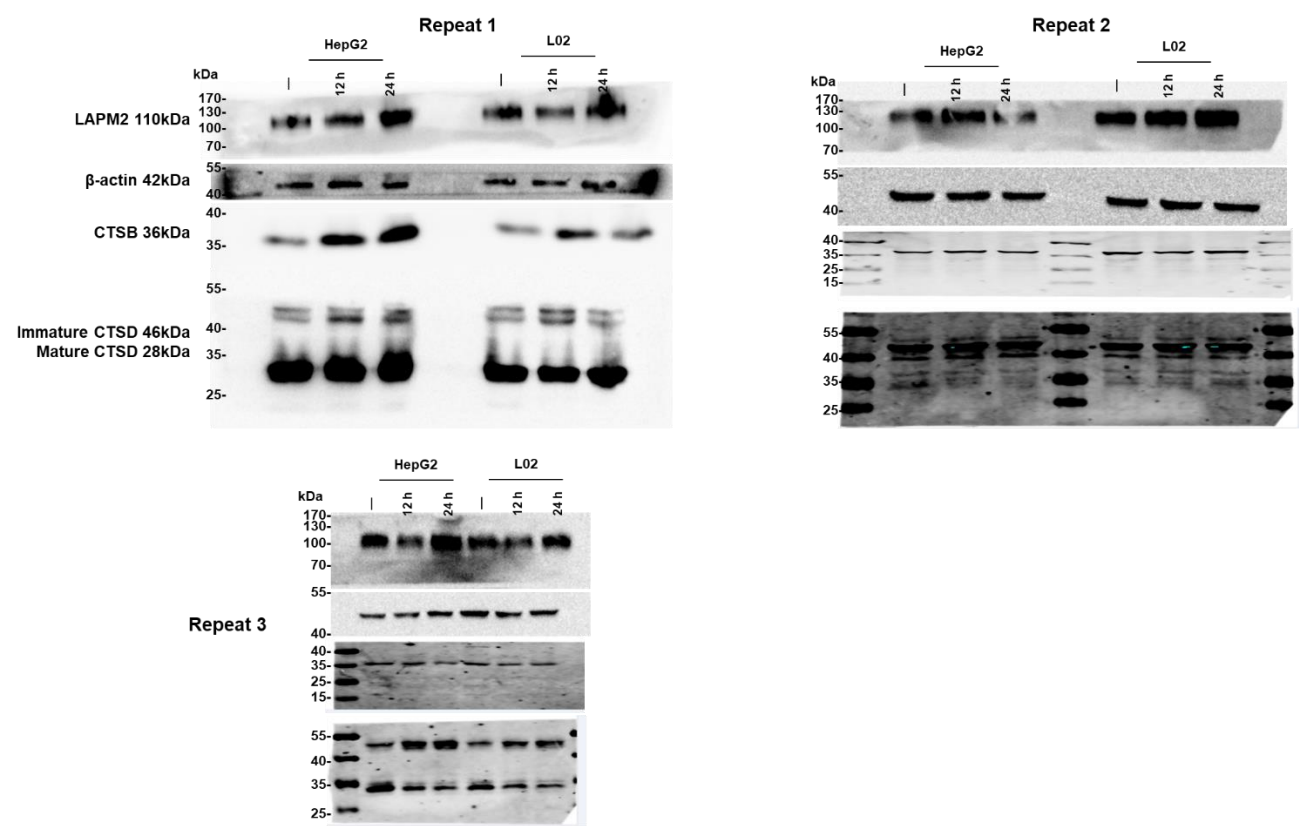

**Figure 4.**

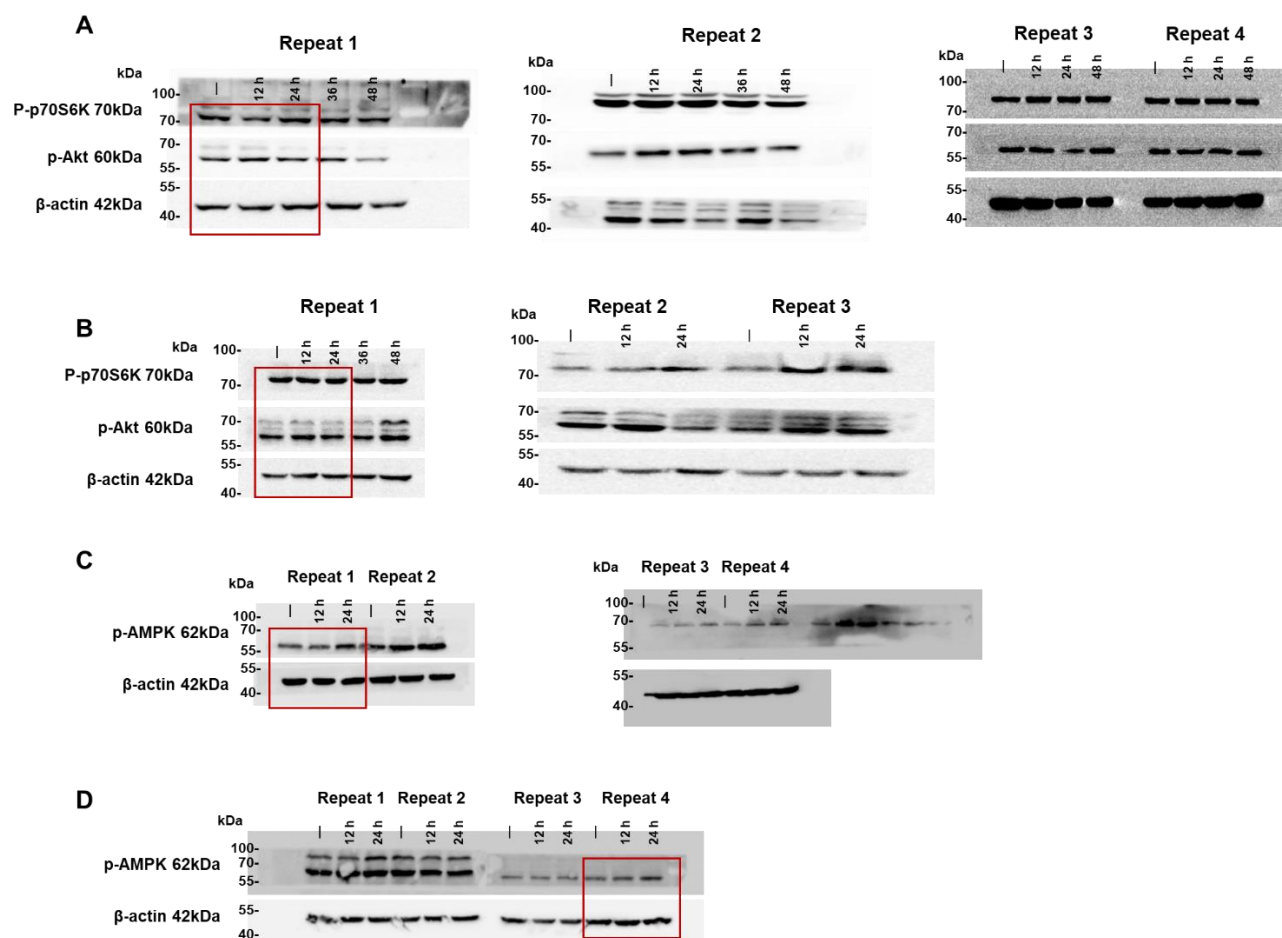

Figure 7.

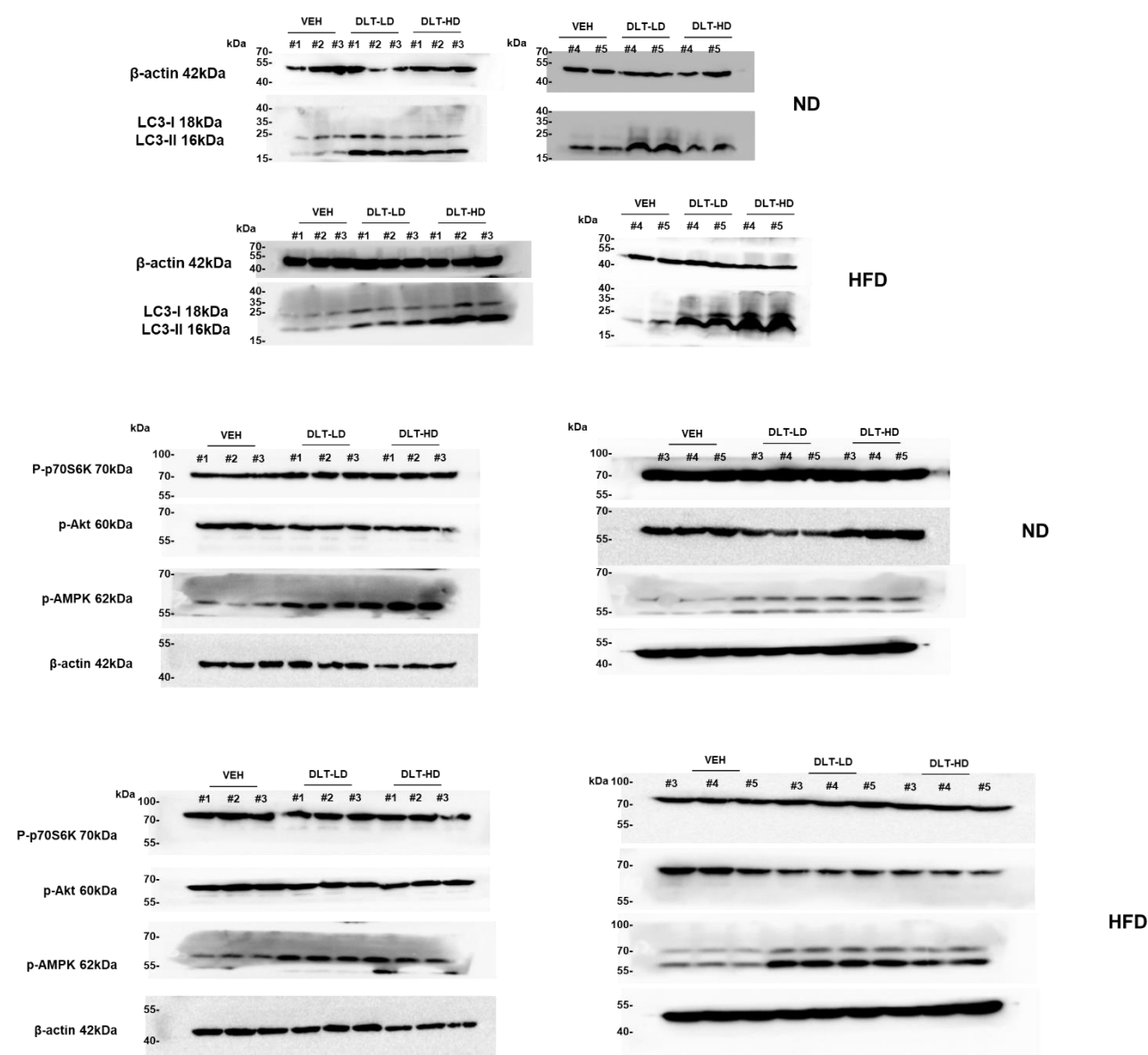

Supplement: Supplementary file 1 [file DataSheet1.PDF]
